# The Impact of Photorespiratory Glycolate Oxidase Activity on *Arabidopsis thaliana* Leaf Soluble Amino Acid Pool Sizes during Acclimation to Low Atmospheric CO_2_ Concentrations

**DOI:** 10.3390/metabo11080501

**Published:** 2021-07-30

**Authors:** Younès Dellero, Caroline Mauve, Mathieu Jossier, Michael Hodges

**Affiliations:** 1 Institute for Genetics, Environment and Plant Protection (IGEPP), National Institute for Research for Agriculture, Food and Environment (INRAE), Institut Agro, Univ Rennes, 35653 Le Rheu, France; 2 Institute of Plant Sciences Paris-Saclay (IPS2), Université Paris-Saclay, National Committee of Scientific Research (CNRS), National Institute for Research for Agriculture, Food and Environment (INRAE), Université d’Evry, Université de Paris, 91190 Gif-sur-Yvette, France; caroline.mauve@ips2.universite-paris-saclay.fr (C.M.); mathieu.jossier@universite-paris-saclay.fr (M.J.)

**Keywords:** acclimation, amino acid metabolism, *Arabidopsis thaliana*, glycolate oxidase, low CO_2_, photorespiration

## Abstract

Photorespiration is a metabolic process that removes toxic 2-phosphoglycolate produced by the oxygenase activity of ribulose-1,5-bisphosphate carboxylase/oxygenase. It is essential for plant growth under ambient air, and it can play an important role under stress conditions that reduce CO_2_ entry into the leaf thus enhancing photorespiration. The aim of the study was to determine the impact of photorespiration on *Arabidopsis thaliana* leaf amino acid metabolism under low atmospheric CO_2_ concentrations. To achieve this, wild-type plants and photorespiratory glycolate oxidase (*gox*) mutants were given either short-term (4 h) or long-term (1 to 8 d) low atmospheric CO_2_ concentration treatments and leaf amino acid levels were measured and analyzed. Low CO_2_ treatments rapidly decreased net CO_2_ assimilation rate and triggered a broad reconfiguration of soluble amino acids. The most significant changes involved photorespiratory Gly and Ser, aromatic and branched-chain amino acids as well as Ala, Asp, Asn, Arg, GABA and homoSer. While the Gly/Ser ratio increased in all Arabidopsis lines between air and low CO_2_ conditions, low CO_2_ conditions led to a higher increase in both Gly and Ser contents in *gox1* and *gox2.2* mutants when compared to wild-type and *gox2.1* plants. Results are discussed with respect to potential limiting enzymatic steps with a special emphasis on photorespiratory aminotransferase activities and the complexity of photorespiration.

## 1. Introduction

In the light, plants carry out photosynthesis that leads to the fixation of atmospheric CO_2_ into organic matter via the carboxylase activity of ribulose-1,5-bisphosphate carboxylase/oxygenase (Rubisco) [1]. Despite a high affinity for CO_2_ and a low affinity for O_2_, Rubisco can fix atmospheric O_2_. Under the actual atmospheric concentrations of CO_2_ and O_2_ (0.041% CO_2_ versus 21% O_2_), the Rubisco of C3 plants can fix approximately one O_2_ for every three CO_2_ molecules assimilated [2]. In addition to 3-phosphoglycerate (3-PGA), this oxygenase activity also produces 2-phosphoglycolate (2-PG), a toxic metabolic intermediate that inhibits triose phosphate isomerase and sedoheptulose-1,7-bisphosphate phosphatase of the Calvin cycle [3,4]. To cope with this problem, plants metabolize 2-PGA to 3-PGA through photorespiration [5]. This pathway requires enzymes located in chloroplasts, peroxisomes, and mitochondria and ultimately leads to the production of one molecule of 3-PGA from two molecules of 2-PG per cycle. while also liberating a molecule each of CO_2_ and NH_3_, producing and consuming a molecule of NADH and consuming one ATP.

Photorespiration has been seen as a wasteful process since it leads to a potential loss of assimilated carbon and nitrogen and it has an energetic cost since released CO_2_ can diffuse to the chloroplast to be re-assimilated [6] and photorespiratory NH_3_ can be re-assimilated by the glutamine synthase/glutamine:2-oxoglutarate aminotransferase (GS/GOGAT) cycle [7]. However, photorespiration is crucial for plant growth in air as seen from studies of photorespiratory mutants that exhibit various deleterious symptoms including dwarfism, chlorosis, and plant death [8]. However, most photorespiratory mutants can be recovered by increasing atmospheric CO_2_ levels, thus reducing the Rubisco oxygenase activity and 2-PG production [9,10,11,12]. The severity of the photorespiratory phenotypes depends on gene redundancy (multiple genes for one enzymatic step) but also on the metabolic step targeted by the mutation, thereby highlighting the multiple interactions between photorespiration and leaf primary metabolism [8,9]. Photorespiration interacts with photosynthesis, nitrogen assimilation, amino acid metabolism, the tricarboxylic acid cycle and C_1_ metabolism [13]. Photorespiration is a major pathway for the production of Gly and Ser in photosynthetic tissues [14] and it is an important part of plant stress responses [15]. Many environmental stresses lead to stomatal closure that lowers CO_2_ levels within leaf mesophyll cells and thus favors photorespiration. In this context, an increased photorespiratory flux can have a beneficial effect by modulating photoinhibition, as seen in Arabidopsis photorespiratory mutants [16]. However, low leaf internal CO_2_ levels may have important consequences for leaf primary metabolism, and specifically amino acid metabolism, since photorespiration is linked to N-metabolism. A recent study in Arabidopsis showed that a short-term low CO_2_ treatment (4–28 h at 100 ppm) promoted the accumulation of urea cycle intermediates (including arginine and ornithine) and a differential regulation of this pathway at the transcriptional level [17]. Other studies showed that a short-term low CO_2_ treatment (2 h at 140 ppm) induced a broad re-orchestration of carbon allocation to major metabolites (including glucose, organic acids and phenylalanine) in sunflowers [18,19].

Photorespiratory enzyme mutants have highlighted the importance of photorespiration in plant amino acid metabolism as seen from metabolite analyses of plants transferred from a low photorespiratory situation (high CO_2_ concentration in air) to normal photorespiration in air. This transfer led to an increase in Gly and Ser levels in wild-type Arabidopsis rosettes [9] but also in the photorespiratory mutants *hpr1*, *pglp1*, and an artificial microRNAi *amirgox1/2* line [4,9,11,12]. After a 5 h transfer from high CO_2_ to air, increased levels of all amino acid levels except for Glu, Asp and Pro were found in the leaves of *pglp1* plants [4]. Changes in Gln, Glu, Asn, Asp and Arg levels were altered after a one-day transfer from high CO_2_ to air in wild-type, *hpr1* and *pglp1* rosettes [9] while after 5 days most amino acids increased in *amirgox1/2* leaves [12]. In air grown Arabidopsis *hpr1, hpr2*, *hpr3* and *ggat1* plants, many amino acid levels were higher compared to wild-type plants [11,20,21] however *ggat1* rosettes contained very low quantities of Ser when plants were grown either in high CO_2_ or in air [11]. On the other hand, air-grown single *gox1* and *gox2* rosettes retaining 30–45% of wild-type GOX activity showed no growth phenotype and exhibited wild-type amino acid levels and photosynthetic capacities [12] probably because the conversion of Gly to Ser by the glycine decarboxylase complex (GDC) has been reported to be the rate-limiting step of the photorespiratory cycle in ambient air [22]. Conversely, *amirgox1/2* targeting both photorespiratory *GOX* genes [12,23,24,25] had only 5% of wild-type leaf GOX activity and exhibited a very strong dwarfism in air [12].

In this study, wild-type Arabidopsis and single *gox1 and gox2* mutants were compared to determine the impact of photorespiration on amino acid metabolism under artificial low atmospheric CO_2_ concentrations. Although such conditions no longer exist on Earth since the last glacial period (18,000–20,000 years ago) when atmospheric CO_2_ levels dropped to 180–190 ppm [26], low internal leaf CO_2_ concentrations are found under certain environmental stress conditions like drought [27]. Short and long-term low CO_2_ treatments were both performed and net CO_2_ assimilation rates, leaf GOX activities and amino acid contents were measured. Statistical analyses revealed significant modifications of amino acid pools following low CO_2_ treatments. There was a specific over-accumulation of Gly and Ser in *gox1* and *gox2.2* mutants, although the Gly/Ser ratio remained similar to that of wild-type Arabidopsis. The metabolic origin of Gly and Ser in the single *gox* mutants under low CO_2_ conditions is discussed.

## 2. Results

### 2.1. Impact of a Short-Term Low CO_2_ Treatment on Photosynthesis and Amino Acid Metabolism in the ARABIDOPSIS gox1 Mutant

**Figure 1:**
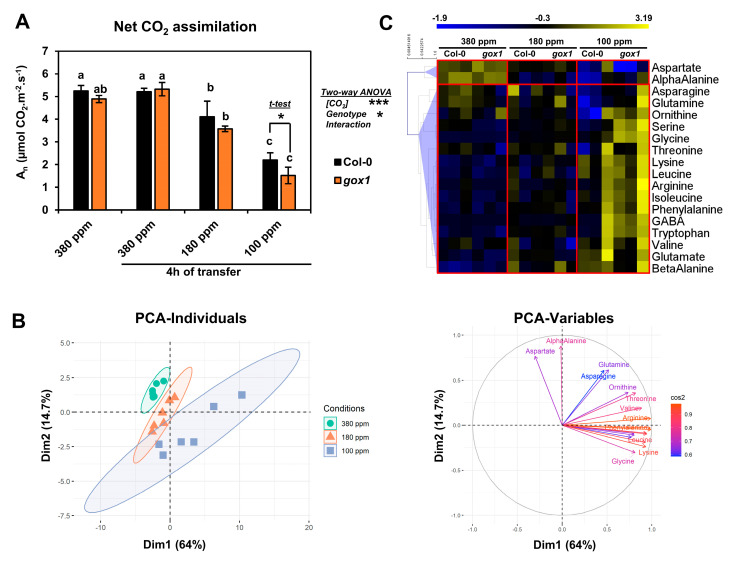
The effect of short-term low CO_2_ treatments (180 and 100 ppm for 4 h) on net CO_2_ assimilation rate and amino acid contents of Arabidopsis Col-0 and *gox1* rosette leaves. (**A**) Net CO_2_ assimilation rate before (380 ppm) and after exposure to an atmosphere with 380, 180 or 100 ppm CO_2_ (4 h treatment). (**B**) Principal Component Analysis of amino acid contents of Col-0 and *gox1* rosette leaves after a 4 h exposure to an atmosphere with 380, 180 or 100 ppm CO_2_. (**C**) Hierarchical clustering of amino acid contents based on Pearson’s correlation coefficients. Results are expressed as the mean ± standard deviation of three independent biological replicates. Different letters indicate groups of mean values that are significantly different between the conditions (two-way ANOVA test followed by a post-hoc Tukey HSD test, *p*-value < 0.05). Ellipses on the PCA plots represent the 95% confidence intervals. *** *p*-value < 0.001; * *p*-value < 0.05.

In an initial short-term low CO_2_ treatment experiment, a small home-made gas exchange chamber was used to compare wild-type Col-0 and *gox1* mutant Arabidopsis lines. The chamber allowed the simultaneous analysis of net CO_2_ assimilation rate of three 5-week-old Arabidopsis plants. After growth in ambient air (380 ppm CO_2_), plants were transferred for 4 h at either 380 ppm (control condition) or at two low CO_2_ concentrations (180 and 100 ppm). Net CO_2_ assimilation rate was not significantly different between wild-type Col-0 and *gox1* either before the transfer of after a transfer to ambient CO_2_ (380 ppm) (Figure 1A). However, there was a gradual and significative decrease in net CO_2_ assimilation rate when low CO_2_ concentrations were applied. The comparison of wild-type Col-0 and *gox1* after 4 h at 100 ppm CO_2_ revealed an additional 30% decrease in net CO_2_ assimilation rate in *gox1* (Figure 1A). A rapid inhibition of photosynthesis in photorespiratory mutants is associated with a limitation of photorespiratory recycling [9,11,12]; therefore, this appeared to have occurred in *gox1* during the 100 ppm CO_2_ treatment.

The same experimental set-up was used with wild-type Col-0 and *gox1* to analyze the contribution of photorespiration to amino acid metabolism under low CO_2_ conditions by quantifying the amino acid content of their rosettes by HPLC (Table S1). From these data, a principal component analysis (PCA) succeeded to separate the three CO_2_ conditions with good confidence (see 95% ellipse confidence on the PCA-Individuals plot of Figure 1B), but not the Col-0 and *gox1* groups. The two first components explained 78.7% of the variance between all samples: Dim1 with 64% and Dim2 with 14.7% (Figure 1B). The contribution of amino acid variations to Dim1 and Dim2 (with correlation circle, Figure 1B) showed that almost all amino acids were correlated with decreased CO_2_ concentration [CO_2_] (Dim1), while Asp and α-Ala were correlated with increased [CO_2_] (Dim2). Only Gln, Asn, Orn and Thr were essentially associated with the variability of biological replicates. A supervised partial least squares.

**Figure 2:**
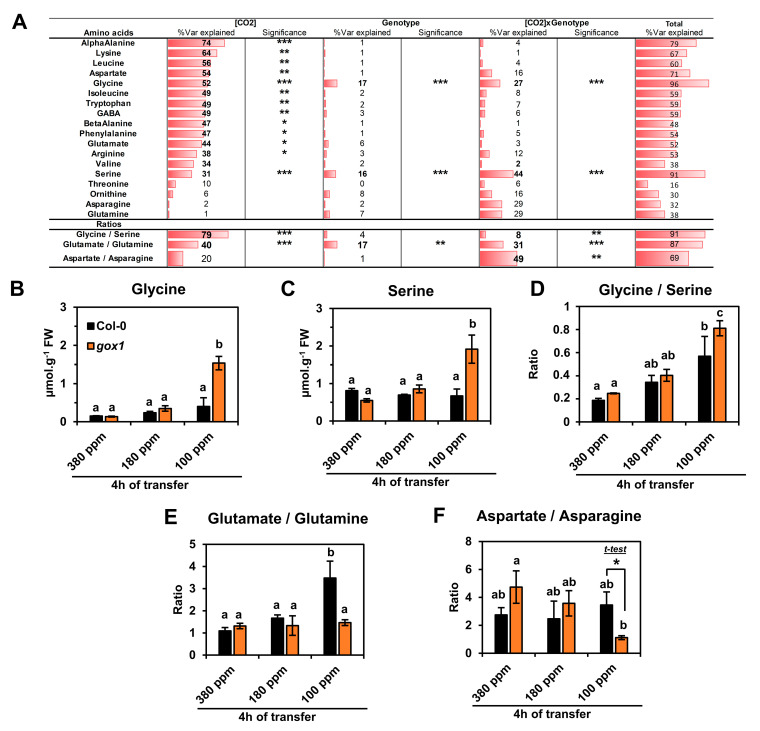
Statistical analyses of amino acid variations associated with short-term exposure to low CO_2_ (180 and 100 ppm for 4 h) in wild-type and *gox1 Arabidopsis thaliana* Col-0. (**A**) Two-way ANOVA analysis of amino acid contents from rosette leaves of Col-0 and *gox1* plants after 4 h of exposure to an atmosphere with 380, 180 or 100 ppm CO_2_. (**B**) Glycine and (**C**) Serine contents, (**D**) Glycine/Serine, (**E**) Glutamate/Glutamine and (**F**) Aspartate/Asparagine ratios. Results are expressed as the mean ± standard deviation of three independent biological replicates. Different letters indicate groups of mean values that are significantly different between the different conditions (two-way ANOVA test followed by a post-hoc Tukey HSD test, *p*-value < 0.05). *** *p*-value < 0.001; ** *p*-value < 0.01; * *p*-value < 0.05. Var = Variance.

Discriminant analysis (PLS-DA) was also performed to identify amino acid variations maximizing the separation of Col-0 and *gox1* groups (Figure S1). However, the results were relatively similar to those of the PCA analysis. Next, a hierarchical clustering analysis was carried out based on Pearson’s correlation coefficients with a heat map representation of normalized-mean center values (Figure 1C). This indicated that Asp and α-Ala contents were strongly decreased at low [CO_2_] (variations of up to 3–4 SD) while many amino acids had their levels increased with decreasing [CO_2_] (variations of up to 4–5 SD). The statistical significance of these results was then assessed using a two-way ANOVA test by considering two factors ([CO_2_] and genotype) and their interaction (Figure 2A). This showed that the variation of Thr, Orn, Asn, Gln and Val was not significantly explained by either [CO_2_] or genotype, thus statistically confirming the PCA analysis. Conversely, large proportions of the variance associated with the other amino

Acids detected were significantly explained by [CO_2_] (total explained Var (%) ranging from 31% to 74%). These observations included notably major plant amino acids (Glu, Asp), aromatic amino acids derived from the shikimate pathway (Phe, Trp), branched-chain amino acids (Leu, Ile), N-rich Arg associated with the urea cycle and photorespiratory amino acids (Gly, Ser). Interestingly, only the variation of Gly and Ser was significantly explained by the genotype (17% and 16% respectively) and the interaction [CO_2_]xGenotype (27% and 44% respectively), leading to the highest levels of total explained variances (96% and 91%, respectively). A detailed survey of Gly and Ser revealed an accumulation in the *gox1* mutant at 100 ppm compared to the control Col-0 (Figure 2B,C; Table S1). Interestingly, the Gly/Ser ratio significantly increased with decreased [CO_2_] (Figure 2D; Table S1) and part of its variance was also explained by the interaction [CO_2_]xGenotype, given the differences observed for Col-0 and *gox1* at 100 ppm (Figure 2A,D). The Asp/Asn and the Glu/Gln ratios were also calculated since they are good indicators of NH_3_ assimilation status by the GS/GOGAT cycle and asparagine synthetase [28,29]. Statistical analyses revealed that a large proportion of the Glu/Gln variability between samples was significantly explained by [CO_2_], the genotype and the interaction [CO_2_]xGenotype (Figure 2A). The Glu/Gln ratio increased with decreased [CO_2_] only in wild-type Col-0 rosettes due to a decrease in Glu (that was significantly different at 100 ppm CO_2_) and an increase in Gln (Figure 2E, Table S1). On the other hand, CO_2_ levels did not significantly affect Glu and Gln levels of *gox1* rosettes (Figure 2E, Table S1). Conversely, the Asp/Asn ratio was significantly decreased in the *gox1* mutant after 4 h at 100 ppm of CO_2_ (Figure 2A,F), and this was due to a reduction in Asp under low CO_2_ conditions compared to ambient air (Table S1).

Overall, a short-term low CO_2_ treatment decreased photosynthetic CO_2_ assimilation and triggered a broad reconfiguration of amino acid metabolism in both control and *gox1* plants. This reconfiguration was more prominent at 100 ppm CO_2_. The remaining GOX activity in *gox1* mutants became rate-limiting for photorespiration based on the increased inhibition of net CO_2_ assimilation rate (photosynthesis) in *gox1* rosettes after 4 h at 100 ppm CO_2_ (Figure 1A). Interestingly, in these conditions, the limitation of GOX activity in *gox1* resulted in an accumulation of Gly and Ser when compared to wild-type Col-0 and this was accompanied by a significant difference of the Gly/Ser ratio between the two lines at 100 ppm of CO_2_ (Figure 2B–D).

### 2.2. Impact of a Long-Term Low CO_2_ Treatment on Amino Acid Metabolism in gox1 and gox2 Mutants

**Figure 3:**
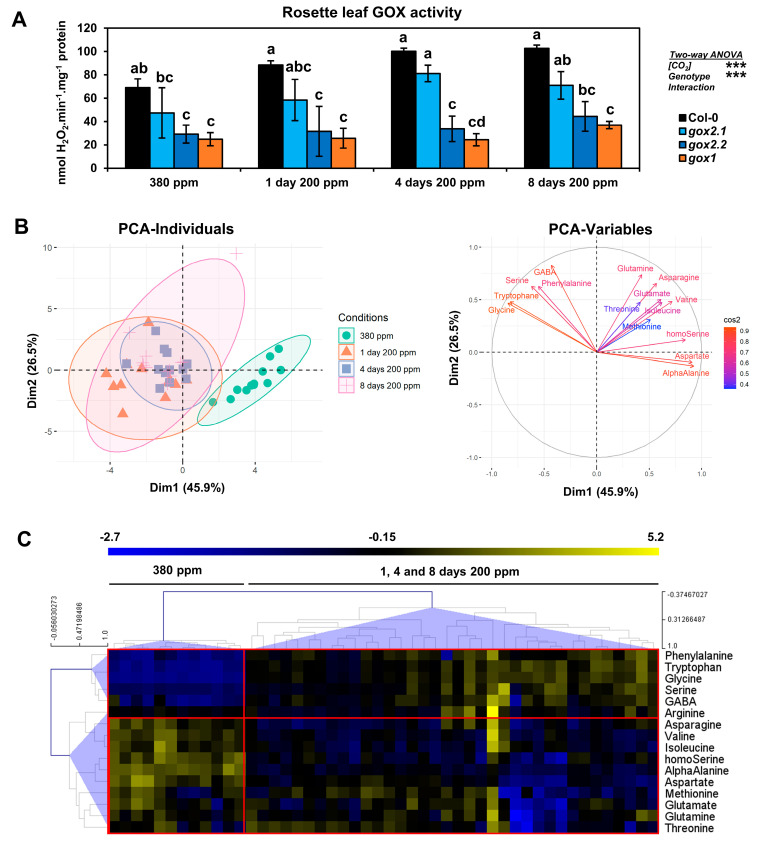
The effect of a long-term low CO_2_ treatment (200 ppm) on rosette leaf GOX activity and amino acid levels of Arabidopsis Col-0, *gox1* and *gox2* mutants. Plants were exposed to an atmosphere containing 200 ppm CO_2_ for either 1, 4 or 8 days. (**A**) Rosette leaf GOX activity, (**B**) principal component analysis of amino acid contents, (**C**) hierarchical clustering of amino acid contents based on Pearson’s correlation coefficients. Results are expressed as the mean ± standard deviation of three independent biological replicates. Different letters indicate groups of mean values that are significantly different between the different conditions (two-way ANOVA test followed by a post-hoc Tukey HSD test, *p*-value < 0.05). Ellipses on PCA plots represent the 95% confidence intervals. *** *p*-value < 0.001.

Given the short-term low CO_2_ condition results, it was decided to analyze the consequences of long-term low CO_2_ conditions on leaf amino acid contents and the contribution of photorespiration to acclimation processes by comparing wild-type Col-0 and several *gox* mutants. Since it was not possible to use the home-made gas exchange system for long-term low CO_2_ treatments, experiments were carried out in a controlled growth cabinet where it was only possible to decrease [CO_2_] to 200 ppm. However, this allowed the testing of additional Arabidopsis *gox* mutant lines; two previously characterized Arabidopsis T-DNA insertion lines namely *gox2.1*, a knock-down line and *gox2.2,* a knock-out line [12]. The three *gox* lines were shown previously to have different levels of leaf GOX activity [12] and therefore a correlation between modifications of amino acid contents observed at low CO_2_ conditions and GOX activity, and subsequently the limitation of photorespiratory glyoxylate production, could be tested. The long-term low CO_2_ experiments were conducted for up to 8 days and rosette leaves were harvested either before (380 ppm) or after 1, 4 and 8 days of transfer to 200 ppm CO_2_. Prior to amino acid analyses, rosette leaf GOX activities were measured to confirm that the mutant lines exhibited different GOX activities during the time-course of the treatments. As expected, at 380 ppm of CO_2_, *gox2.1* plants had around 69% of wild-type GOX activity while *gox2.2* and *gox1* had 42% and 36%, respectively (Figure 3A). GOX activity was significantly increased for all lines after the transfer to 200 ppm [CO_2_], but this had no significant impact on GOX activity differences between the genotypes (no significant interaction [CO_2_]xGenotype from the two-way ANOVA test).

**Figure 4:**
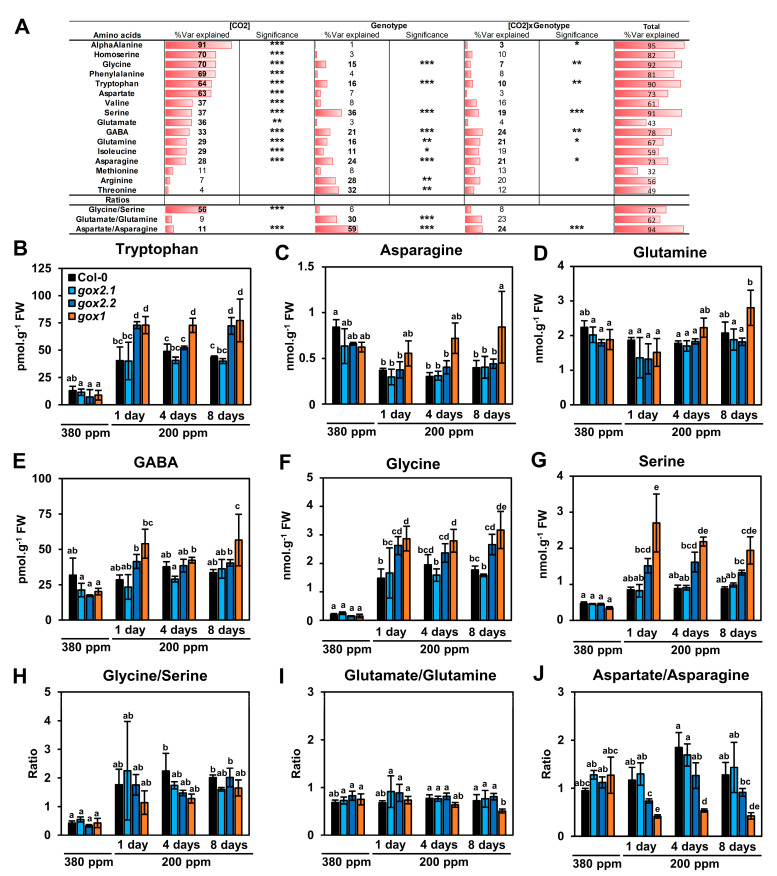
Statistical analyses of amino acid variations associated with long-term low CO_2_ treatment (200 ppm) in *gox1* and *gox2* mutants. (**A**) Two-way ANOVA analysis of amino acid contents from rosette leaves of Col-0, *gox1* and *gox2* mutants after 1, 4 or 8 days of exposure to an atmosphere with 200 ppm of CO_2_. (**B**) Tryptophan, (**C**) asparagine, (**D**) glutamine, (**E**) GABA, (**F**) glycine and (**G**) serine contents. Ratios for (**H**) glycine/serine, (**I**) glutamate/glutamine, (**J**) aspartate/asparagine. Results are expressed as the mean ± standard deviation of three independent biological replicates. Different letters indicate groups of mean values that are significantly different between the different conditions (two-way ANOVA test followed by a post-hoc Tukey HSD test, *p*-value < 0.05). *** *p*-value < 0.001; ** *p*-value < 0.01; * *p*-value < 0.05. Var = Variance.

Amino acid contents of wild-type Col-0 rosette leaves and of each *gox* mutant were quantified before (380 ppm) and after 1, 4 and 8 days of transfer to 200 ppm CO_2_ (Table S2). A PCA was carried out and the two first components explaining the maximum variance between all samples were selected: Dim1 with 45.9% and Dim 2 with 26.5% (Figure 3B). Again, this only separated the 380 ppm and 200 ppm CO_2_ conditions with a good confidence (see 95% ellipse confidence on the PCA–Individuals plot of Figure 3B). The contribution of amino acid variations to Dim1 and Dim2 (Figure 3B) showed that Gly, Ser, Trp, GABA and Phe were highly correlated with the separation of 380 and 200 ppm CO_2_ conditions (combination of both Dim1 and Dim2) while Asp, α-Ala and homoSer were negatively correlated with decreased [CO_2_] (mainly Dim1). A supervised PLS-DA was performed to improve the identification of amino acids whose variations maximized the separation of Col-0, *gox2.1*, *gox2.2* and *gox1* mutant groups. However, the results were relatively similar to those of the PCA analysis (Figure S2). A hierarchical clustering analysis based on Pearson’s correlation coefficients defined two groups of amino acids (Figure 3C). The first group comprised some amino acids also identified by the PCA analysis (notably Phe, Trp, Gly, Ser and GABA) where their contents were strongly increased after the transfer from 380 ppm to 200 ppm CO_2_ (variations from for to seven SDs). The second group comprised amino acids that exhibited decreased contents after transfer from 380 ppm to 200 ppm CO_2_ and included Asp, α-Ala and homoSer (variations from three to six SDs). The statistical significance of these results was assessed using a two-way ANOVA test by considering the two factors “[CO_2_]” and “genotype” and their interaction (Figure 4A). Similarly to the short-term transfer to low [CO_2_], the variation of many amino acids was significantly explained by [CO_2_], except for Met, Arg and Thr. However, a proportion of amino acid variability was also explained by the genotype and the [CO_2_]xGenotype interaction. This was most notable for Gly, Trp, Ser, GABA, Gln and Asn (Figure 4B–G). Globally, many of the differences observed between the mutant lines and the control after their transfer occurred from day 1 and remained relatively stable until day 8, as previously suggested by PCA and hierarchical clustering (Figure 3). Interestingly, Trp and GABA contents increased more in the two knock-out mutants *gox1* and *gox2.2* compared to wild-type Col-0 and the knock-down line *gox2.1* after transfer to 200 ppm CO_2_ (Figure 4B,E). For Asn and Gln, an increase was essentially observed only in the *gox1* mutant, and suggested that N assimilation was more affected by the transfer in this mutant compared to the other lines (Figure 4C,D). As observed with the short-term low CO_2_ treatment, Gly and Ser contents were significantly increased in the control line following transfer from 380 ppm to 200 ppm CO_2_ (Figure 4F,G). Although there was a higher accumulation of Gly and Ser in *gox1* and *gox2.2* compared to the control and *gox2.1*, a similar increase in the Gly/Ser ratio from 0.4 to around 2 was observed for all lines after transfer to low CO_2_ (Figure 4A,H). The short-term low CO_2_ treatment led to modifications of Asp, Asn, Gln and Glu amounts depending on the genotype (Figure 4C,D and Table S1) and this impacted both Asp/Asn and Glu/Gln ratios (Figure 4A,I,J). Statistical analyses revealed that 30% of the Glu/Gln variability was significantly explained by the genotype while 11%, 59% and 24% of the Asp/Asn variability was significantly explained by [CO_2_], genotype and the [CO_2_]xGenotype interaction, respectively. The Glu/Gln ratio significantly increased with respect to wild-type Col-0 only in the *gox1* mutant after 8 days of transfer to 200 ppm CO_2_ while the Asp/Asn ratio was significantly increased in both *gox1* and *gox2.2* in low CO_2_.

## 3. Discussion

**Figure 5:**
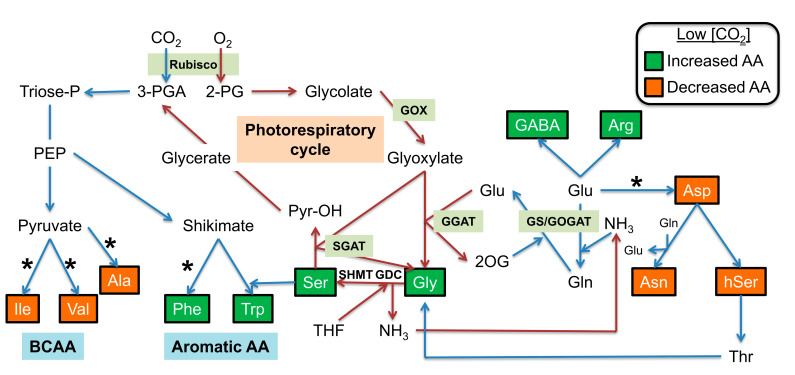
Simplified metabolic scheme highlighting low-CO_2_ induced changes in amino acids. In low CO_2_ conditions, Rubisco oxygenase activity is increased at the expense of Rubisco carboxylase activity. This led to increased (green boxed) and decreased (orange boxed) soluble amino acid levels in Arabidopsis rosette leaves. Photorespiratory NH_3_ is reassimilated to produce Glu via the GS/GOGAT cycle and this Glu is recycled by the glutamate:glyoxylate aminotransferase (GGAT). Glyoxylate produced by glycolate oxidase (GOX) is a common substrate of GGAT and the serine:glyoxylate aminotransferase (SGAT). * Indicates Glu-dependent aminotransferases involved in amino acid biosynthesis pathways. Other abbreviations: 2OG, 2-oxoglutarate; 2-PG, 2-phosphoglycolate; 3-PGA, 3-phosphoglycerate; AA, amino acids; BCAA, branched-chain amino acids; GDC, glycine decarboxylase; GOGAT, Gln:2-oxoglutarate aminotransferase; GS, glutamine synthetase; PEP, phosphoenolpyruvate; Pyr-OH, hydroxypyruvate; Rubisco, ribulose-1,5-bisphosphate carboxylase/oxygenase; SHMT, serine hydroxymethyl transferase; THF, tetrahydrofolate; Triose-P, triose-phosphates.

Photorespiration is an essential metabolic pathway in air-grown plants but it limits plant productivity [30]. It also plays a preponderant role in plant resistance to abiotic and biotic stresses [15,16,31,32,33]. Under such stress conditions, stomatal closure results in low CO_2_ concentrations at the vicinity of Rubisco thus reducing photosynthesis and promoting photorespiration and therefore impacting both carbon and nitrogen metabolisms. In such situations, higher photorespiration will impact both carbon and nitrogen metabolisms due to increased demands for the re-assimilation of photorespiratory-released NH_3_ and CO_2_. To investigate the impact of low CO_2_ concentrations and photorespiration on amino acid metabolism, Arabidopsis single *gox* mutants and short- and long-term transfers of plants from air to low CO_2_ conditions were used to modulate photosynthesis and photorespiration. To date, the impact of photorespiration on plant metabolism has often been studied using photorespiratory mutants and their transfer from high CO_2_ (for example 3000 ppm CO_2_, low photorespiration) to ambient air (400 ppm CO_2_, normal photorespiration) (for a review see [8]). The effects of low CO_2_ concentrations on plant metabolism have been less studied, perhaps due to practical reasons to maintain low CO_2_ air. Nevertheless, it has been reported that low CO_2_ induces the accumulation of urea cycle intermediates [17] and short-term low CO_2_ treatments were used to study Rubisco oxygenase activity (v_0_) and amino acid metabolism. The latter highlighted negative correlations between v_0_ and Ala and Asp while a positive correlation was reported between v_0_ and Gly and Ser [34]. Short-term low CO_2_ treatments coupled to ^13^C-labelling have been used to examine changes in photosynthesis and photorespiration on plant metabolism including recently the in vitro stoichiometry of photorespiratory metabolism [35], carbon allocation to major metabolites [18], the metabolic origin of carbon atoms in glutamate [36] and in vivo phosphoenolpyruvate carboxylase activity [37]. In this work, both short-term (4 h) and long-term (1 to 8 d) low CO_2_ treatments led to changes in amino acid pool sizes (see Tables S1 and S2). In general, maximal changes were attained after one day at low CO_2_ after which they remained stable. This shows that plant acclimation to low CO_2_ leads to a relatively rapid new metabolic homeostasis (Figure 3 and Figure 4; Table S2). This was associated with changes in photorespiratory enzyme capacity as seen from rosette GOX activities (Figure 3A). Overall, the long-term 200 ppm CO_2_ treatment triggered both depletions (Ala, Asn, Asp, homoSer, Ile and Val) and augmentations (Gly, Ser, Phe, Trp, Arg and GABA) in amino acid pool sizes (Figure 5, Table S2) while some changes were also photorespiratory-genotype dependent (Figure 4, Table S2). Interestingly, amino acid changes observed after short-term low CO_2_ treatments (Figure 2, Table S1) were more pronounced in the *gox1* mutant and after transfer of wild-type Col-0 to 100 ppm CO_2_ (Table S1). A small number of differences in amino acid accumulations/depletions between the different CO_2_ conditions were observed, especially with respect to branched-chain amino acids (compare Tables S1 and S2). This could be due to specific responses depending either on the level of the low CO_2_ treatment and/or a temporal modulation of this response. Genotype differences (wild-type Col-0 v *gox* mutants) were associated with *gox* mutants having the lowest GOX activities (*gox1* and *gox2.1*) (Figure 4, Table S2). This observation suggested an additional photorespiratory-dependent effect that could be due to limited glyoxylate production under higher photorespiration conditions.

Low CO_2_ acclimation did not appear to bring about changes associated with carbon starvation such as an increase in total amino acid pools due to protein degradation [12,38]. During both short- and long-term CO_2_ treatments, total soluble amino acid amounts remained relatively stable except in the *gox1* and *gox2.2* mutant lines due to a much higher accumulation of photorespiratory Gly and Ser compared to wild-type Col-0 and *gox2.1* lines (Figure S3; Tables S3 and S4). This was also observed between *gox1* and wild-type Col-0 after a short-term transfer to 100 ppm CO_2_. Therefore, the low CO_2_-induced over-accumulation of Gly and Ser in the *gox* mutants was related to

Their extractable leaf GOX activities and this will be discussed below (see Section 3.3). However, not only photorespiratory-associated amino acids were affected by a low CO_2_ atmosphere. Both low CO_2_ treatments resulted in the depletion of Ala and Asp in all lines thus identifying them along with Gly and Ser as important markers of low CO_2_/high photorespiration (Figure 1 and Figure 3; Tables S1 and S2). In addition to these major amino acids, some minor amino acid pools including Arg, branched-chain and aromatic amino acids were also modulated by low CO_2_ (Figure 1, Figure 3 and Figure 5; Tables S1 and S2). It might be expected that under low CO_2_ conditions, less carbon is available for N-assimilation and amino acid biosynthesis thereby decreasing amino acid pool sizes. This was clearly not the case since a number of amino acid pool sizes increased. This can happen because either they are being used less or being produced more. It should be noted that variations of amino acid pool sizes are clearly not always correlated with intuitive variations of their associated metabolic fluxes [39].

### 3.1. Minor Amino Acids: Arg, Aromatic and Branched-Chain Amino Acids under Low CO_2_ Conditions

Minor amino acid pools modulated by low CO_2_ conditions represented a very small percentage of the total soluble leaf amino acid pool (each < 2%) (Figure S3; Tables S3 and S4), thus representing very small carbon and nitrogen sinks. A previous study reported an accumulation of Arg and Orn in Arabidopsis after transfer from 400 ppm to 100 ppm CO_2_ [17] and this was explained by an excess of re-fixed photorespiratory N under high photorespiratory conditions being stored in N-rich amino acids due to carbon limitations [17]. In these experiments, net CO_2_ assimilation rates were similar to values obtained in the short-term experiments that led to an accumulation of Arg after 4 h at 100 ppm CO_2_ especially in *gox1* rosettes and to a lesser extent in wild-type Col-0 (Figure 1A,C; Table S1). However, after transfer to 200 ppm CO_2_, Arg levels only significantly increased in *gox1* rosettes while wild-type Col-0 and gox2.2 Arg levels appeared to decrease 1 and 4 days after transfer (Figure 3C; Table S2). Thus, the observed low CO_2_ Arg biosynthesis depended on both altered CO_2_ assimilation rates and photorespiratory capacities. This was accentuated when photorespiratory cycle functioning was altered in certain *gox* mutants leading to a further augmentation of carbon sequestration in photorespiratory Gly and Ser (Figure 4G) and/or an inhibition of Calvin cycle RuBP regeneration by the accumulation of 2-PG [4]. Such situations could have provoked a C-starvation syndrome as proposed in [17].

Low CO_2_ conditions also led to an accumulation of Phe and Trp (Figure 1C and Figure 3C; Tables S1 and S2), both aromatic amino acids produced via the shikimate pathway and requiring PEP and erythrose-4-phosphate as C-skeletons (Figure 5). A short-term CO_2_ effect was more significant at 100 ppm in the *gox1* mutant line and only for Trp (Table S1) while the long-term, low CO_2_ treatment brought about significant increases of both Phe and Trp in all genotypes although the effect was greater in *gox1* and *gox2.2* plants (Figure 4B, Table S2). It is tempting to propose a stimulated shikimate pathway under low CO_2_ conditions, but this would be unexpected due to the reduced photosynthetic activity producing fewer C-skeletons. A recent study found that the incorporation of photosynthetically neo-assimilated carbon towards Phe biosynthesis was reduced by up to 35% following a transfer from 380 to 140 ppm CO_2_ using ^13^CO_2_ labeling [19]. This clearly shows a decrease in de novo biosynthesis of Phe under low CO_2_ conditions and therefore intuitionally the observed increase in Phe pool size probably reflected a reduced utilization.

Carbon-starvation in plants often occurs during stress conditions and induced protein degradation that generates an increase in branched-chain amino acids that are used for energy production via mitochondrial respiration [40,41,42]. Inhibition of photosynthesis in the photorespiratory mutant *amiRgox1/2* triggered such symptoms after transfer from high CO_2_ (3000 ppm) to normal air [12]. It might be that the accumulation of Leu, Ile and Val at 100 ppm CO_2_ was brought about by a rapid C-starvation that either stimulated BCAA biosynthesis or reduced BCAA consumption. However, this was not the case during a long-term low CO_2_ treatment. In this situation, the observed reduction in BCAA levels could simply reflect lower photosynthetic carbon assimilation leading to a reduction in their biosynthesis. This agrees with the reduced incorporation of photosynthetically neo-assimilated carbon towards Val following a transfer from 380 to 140 ppm CO_2_ [19]. Nevertheless, the use of BCAAs as supplementary respiratory substrates cannot be excluded since glycolytic-derived substrates would be expected to be negatively impacted by less CO_2_ assimilation under low CO_2_ compared to 380 ppm CO_2_ air.

### 3.2. Major Amino Acids under Low CO_2_ Conditions

In 380 ppm CO_2_ air, major soluble amino acids included Gln, Glu, Asn, Asp and Ala (see Tables S3 and S4). The importance of Glu and Gln for N-assimilation via the GS/GOGAT cycle is highlighted by the observation that low CO_2_ acclimation did not significantly alter the Glu/Gln balance and their individual pool sizes (Figure 4I, Table S2). This observation agrees with an absence of a correlation between v_0_ with Glu and Gln levels and their stability in potato and wheat [34]. Since photorespiratory ammonium reassimilation and the GS/GOGAT cycle are linked by a cycling of Glu (Figure 5), this would require the maintenance of an adequate Glu supply especially under conditions where N is being sequestered in Gly and Ser (Figure 2 and Figure 3; Tables S3 and S4; see Section 3.3). This could be achieved by either increasing primary N-assimilation [43] and/or modulating the biosynthesis of other major amino acids derived from Glu. In agreement with this scenario, low CO_2_ acclimated plants contained lower levels of Ala, Asp and BCAAs when compared to plants grown in 380 ppm CO_2_ (Figure 1 and Figure 3; Tables S1 and S2). The biosynthesis of these amino acids involves Glu-dependent aminotransferase reactions (Figure 5). Due to lower CO_2_ assimilation rates at low CO_2_, the reduction of Ala and Asp could simply reflect reduced amounts of pyruvate and oxaloacetate required for transaminase activities. Furthermore, Asp could be consumed to maintain the malate/OAA balance allowing photorespiratory redox transfer from the mitochondria when photorespiration is high (see [32]). These scenarios would help reduce Glu consumption and help maintain Glu levels. The observed reduction in Asn levels (albeit not seen in the *gox1* mutant for reasons that require further exploration) (Figure 4C) could be the consequence of low Asp levels while a lower asparagine synthetase activity would also help maintain Gln levels. Decreases in Ala and Asn could also be due to their increased use by photorespiratory SGAT [44]. GABA is also linked to Glu metabolism, and it increased under low CO_2_ but only in *gox1* and *gox2.2* mutants. GABA transferase converts GABA to succinic-semialdehyde and it uses either pyruvate or glyoxylate as amine acceptor to produce alanine and glycine, respectively [45]. Therefore, in these mutants at low CO_2_, GABA transferase activity could be reduced and thus lower GABA catabolism due to insufficient glyoxylate production.

### 3.3. Gly and Ser Become Major Amino Acids under Low CO_2_ Conditions and Accumulate More in Certain gox Mutants

Under normal air CO_2_ conditions, Gly and Ser were not major amino acids and together they represented only 8% of the total soluble amino acid pool (Figure S3). However, after a long-term low CO_2_ treatment they made up 27–61% of the total soluble amino acid pool (depending on genotype) and thus became an important N-sink (Tables S3 and S4). High photorespiration conditions also led to an increase in the Gly/Ser ratio (that has been correlated with v_0_, [34]) from 0.5 to around 2 in all genotypes when transferred to low 200 ppm CO_2_ (Figure 4H) due to larger increases in Gly compared to Ser (Figure 4F–G). The compartmentalization of photorespiration, its cyclic nature and its links with other metabolic pathways make it difficult to predict and to pinpoint the major processes that influence the differential change in Gly and Ser levels. A preferential increase in Gly could be the consequence of the GDC reaction becoming more limiting with respect to the SHMT1 reaction. It could also be influenced by the two aminotransferase reactions carried out by GGAT1 and SGAT1 that both produce Gly and require glyoxylate/Glu and glyoxylate/Ser, respectively. A limiting GDC reaction was suggested from changes in rosette Gly levels of Arabidopsis plants over-expressing either GDC-L [46] or GDC-H [47]. It has been shown also that THF availability for the GDC reaction is important to maintain Ser metabolism [48]. On the other hand, plants over-expressing SGAT1 exhibited very low Ser levels [49] and ^14^C-labelling of *sgat1* mutants showed a much higher incorporation of ^14^C into Ser compared to Gly [10,50]. When a barley *sgat* mutant was transferred to air from high CO_2_ conditions, Ser levels were dramatically increased (40-fold) compared to Gly (only fourfold) [51]. Again, in a barley *sgat* knock-down line with 50% wild-type activity, Ser levels were increased more than Gly [52]. In tobacco, ^14^C-glycolate labeling of a *sgat* mutant led to a high incorporation of ^14^C into Ser whereas Gly was less labeled [50]. Thus, modified SGAT activity appears to modulate preferentially Ser levels compared to Gly. Arabidopsis *ggat1* mutant rosettes containing only 10–20% of wild-type Ser levels, showed only a twofold increase in Gly when transferred from high CO_2_ to air while wild-type plants exhibited a sevenfold increase [11]. On the other hand, Arabidopsis plants over-expressing GGAT1 indicated a positive correlation between GGAT activity and Gly and Ser levels but Ser levels were increased more than Gly [53]. Such observations clearly indicate an important influence of GGAT1 and SGAT activities in determining photorespiratory Gly and Ser levels. They also show that there is often a differential modulation of Gly and Ser that leads to a higher accumulation of Ser. This might be expected when SGAT activity is reduced [51,52] but it is less intuitive when GGAT1 activity is increased [53]. In low CO_2_ conditions, Gly levels increased more than Ser levels (Figure 2 and Figure 4). This could reflect a GDC limitation due to inadequate THF levels and/or a retroinhibition by Ser on GDC activity [54]. This could be coupled to an altered equilibrium between the two aminotransferases due to their differential promiscuity with respect to alternative substrates and their kinetic properties. SGAT is associated with multiple activities while GGAT appears to undertake only two different transaminase reactions [44,55,56]. Although, Arabidopsis GGAT and SGAT have quite similar Km values for glyoxylate (0.21 mM and 0.11 mM) and for their respective amino acids (2 mM and 3 mM) [56,57], the kcat of GGAT was found to be sevenfold faster than that of SGAT (145 s^−1^ versus 20 s^−1^) in rice [58]. Such observations suggest that in planta, the production of Gly via GGAT1 would be more important than the removal of Ser by SGAT and thus lead to a low Gly/Ser ratio when GDC activity is not limiting.

The increase in Gly and Ser was much higher in the *gox1* and *gox2.2* mutants compared to *gox2.2* and wild-type plants when transferred to 200 ppm CO_2_ (Figure 4F–G) and this appeared to be correlated with extractable rosette GOX activities (Figure 3A). As mentioned above, Gly and Ser accumulation is common to many Arabidopsis photorespiratory mutants after their transfer from high CO_2_ (low photorespiration) to ambient air (normal photorespiration). But a higher accumulation is observed in mutants lacking enzymes downstream from the GDC/SHMT step. Arabidopsis *hpr1-1* plants transferred from high CO_2_ to air accumulated 80-fold more Gly and ninefold more Ser after one day while wild-type plants showed 2.5-fold changes in both amino acids [9]. The complexity of the photorespiratory cycle makes it difficult to predict the consequences of a specific mutation on Gly and Ser contents. Arabidopsis *pglp1* mutants accumulated similar amounts of Gly and Ser as wild-type plants after their transfer from high CO_2_ to ambient air, although they already contained 18-fold more than wild-type plants under high CO_2_ [9]. The transfer from high CO_2_ to air of *amiRgox* plants with only 5% wild-type GOX activity brought about a 2.5-fold increase in Gly while a fivefold increase was seen in wild-type plants [12]. It appeared that the metabolic origin of the additional accumulated Gly and Ser in *gox1* and *gox2.2* was due to the low CO_2_ conditions stimulating photorespiration. It might be that in these mutants there is an activation of alternative metabolic routes to make Gly and Ser. For instance, Gly can be synthesized from Thr via Thr aldolase, although this activity is nonessential for Gly production in Arabidopsis under normal conditions [59], and from non-photorespiratory aminotransferases [57,60]. Ser can be made via the phosphoserine pathway that plays an essential role in embryo and pollen development [61,62]. This pathway became important in photosynthetic tissues when photorespiration was reduced [63] and it was induced in plants over-expressing SGAT [49]. However, such scenarios do not make sense in plants that are already accumulating large quantities of both amino acids. It is probably more important for *gox1* and *gox2.2* plants to maintain their capacity to produce glyoxylate, possibly by activating alternative non-photorespiratory pathways. A candidate could be the first enzyme of the glyoxylate cycle [64], isocitrate lyase (ICL) that produces glyoxylate from isocitrate. This cycle generates respiratory substrates from triacylglycerol degradation during seed germination but it is not present after this developmental stage, although ICL is induced by natural senescence [65]. This hypothesis requires further investigation, but it is probably difficult to reconcile such a scenario with Gly and Ser over-accumulation unless ICL allows for a higher glyoxylate production compared to wild-type Col-0 and *gox2.2* plants. On the other hand, a perturbation of glyoxylate metabolism in the *gox1* and *gox2.2* mutants might alter the coordinated action of the two glyoxylate-dependent aminotransferase reactions that link photorespiratory Gly and Ser metabolisms. Based on the kinetic properties of Arabidopsis GGAT and SGAT [56,57], it is difficult to propose a differential effect of glyoxylate concentration on the transaminase activities that could explain the higher accumulation of Gly and Ser in *gox1* and *gox2.2*. So, could it be that photorespiration is actually higher in the *gox1* and *gox2.2* plants due to their lower capacity to produce glyoxylate? Several observations made under non-physiological conditions using very high mM quantities of glyoxylate suggested that high amounts of glyoxylate actually improved photosynthesis, and led to lower amounts of both glycolate and glycine by inhibiting photorespiration [54,66]. Therefore, it is possible that the inverse is occurring in *gox1* and *gox2.2* rosettes where a lower maximal GOX activity reduces steady-state glyoxylate production and this has a beneficial effect on the photorespiratory cycle and allows for the production of more Gly and Ser under a high photorespiration situation (low CO_2_).

## 4. Materials and Methods

### 4.1. Plant Material and Growth Conditions

Experiments were performed using *Arabidopsis thaliana* wild-type ecotype Columbia (Col-0) and previously characterized T-DNA insertion mutants for *GOX1* (*At3g14420*; SAIL 117-G11 (*gox1*)), and *GOX2* (*At3g14415*; Salk 025,574 (*gox2.1*) and Salk 044,052 (*gox2.2*)) [12]. Seeds were germinated for one week and then individually transferred to medium pots for plant growth. The following climatic conditions were used for plant growth: 8 h/16 h day/night cycle (20 °C/18 °C), light intensity of 200 µmol photons.m^−2^.s^−1^, ambient air (380 µmol CO_2_.mol^−1^ air) and a relative humidity of 65–80%. Prior to low CO_2_ exposure, plants were grown for 5 weeks on a commercial peat substrate fertilized with 1 kg.m^−3^ of a 17:10:14 N/P/K mixture and irrigated twice a week with tap water.

### 4.2. Low CO_2_ Exposure Experiments

Two different experiments were performed: a short-term 4 h transfer and a long-term transfer for up to 8 days.

For short-term exposure to low CO_2_, three plants per condition and per genotype were taken from the growth cabinet 1 h after the beginning of illumination and transferred to a home-made gas exchange open system (0.8 dm^3^) illuminated with six LEDs (200 µmol photons.m^−2^.s^−1^) and connected to a portable photosynthesis system (LiCOR 6400XT). The upper part of the gas exchange chamber was built with a breakable soft and transparent film, thus allowing instant freezing of rosette leaves with liquid nitrogen spraying at the end of the experiment. The air flow inside the chamber was kept to around 40 L.h^−1^ with an air pump and the air temperature inside the chamber was kept to around 20 °C by using a coil cooler immersed into a water bath kept at 8 °C. A thermocouple was placed close to a leaf inside the chamber and connected to the LiCOR to follow air temperature evolution during the experiments. Relative humidity (65–80%) and [CO_2_] inside the chamber were directly controlled with the portable photosynthesis system (LiCOR 6400XT). Prior to low CO_2_ treatment, plants were acclimated for up to 1 h in the chamber at 380 ppm CO_2_. The speed of air renewal with this experimental setup allowed rapid adjustment of CO_2_ levels within the chamber (1–3 min).

For long-term exposure to low CO_2_, it was not possible to use the home-made gas exchange system. Therefore, a growth cabinet with a CO_2_ detector was equipped with two large cotton bags full of soda lime (1–2 kg per bag) in front of the air fans located within the chamber. The soda lime was renewed every 2 days and allowed approximately 200 ppm CO_2_ to be reached inside the growth cabinet. The experiment started with the transfer of plants taken from a classic growth cabinet (at 380 ppm) 1 h after the beginning of illumination. Samples were harvested after 1, 4 and 8 days by quickly cutting rosette leaves inside the chamber and putting them into liquid nitrogen (semi-instantaneous freezing).

### 4.3. Amino Acid Quantification

Frozen rosette leaves were ground to a fine powder in a mortar pre-cooled with liquid nitrogen and polar metabolites were extracted with ice-cold methanol/water (80:20) containing 100 µM of α-aminobutyrate as an internal standard. A ratio of 1 mL per 100 mg FW of rosettes was used. After a 15 min centrifugation step at 10,000× *g* at 4 °C, the supernatant was recovered and again subjected to the same centrifugation step. For each supernatant, an aliquot of 200 µL was dried overnight under vacuum and stored at −80 °C. Amino acids were derivatized with o-phthaldialdehyde (OPA), separated by HPLC on a “Symmetry C18 3.5 μm” column (150 mm × 4.6 mm, Waters) and detected with a fluorescence detector as previously described [67]. Three points of a calibration curve made with a mix of amino acid standards were injected every five samples to monitor the drift of the fluorescence response of OPA-derivatives during the run. Amino acid derivatives were identified by comparison of their retention times with authentic standards and quantified using a calibration curve, after a correction step with blank values and a normalization step with the internal standard α-aminobutyrate.

### 4.4. GOX Activity

Frozen rosette leaves were ground to a fine powder and leaf soluble proteins were extracted at 4 °C with 50 mM Tris-HCl, pH 8.0 containing an anti-protease cocktail (Complete-Mini, Roche). After a centrifugation step of 10 min at 20,000× *g* at 4 °C, 500 µL of each supernatant was desalted by filtration using individual ice-cold NAP-5 columns (GE Healthcare). Leaf soluble protein levels of the desalted fractions were quantified using the Bradford reagent (Sigma-Aldrich, St. Louis, MO, USA) with bovine serum albumin as the standard. The desalted extracts were immediately used for GOX activity measurements. The H_2_O_2_ produced by GOX activity was measured spectrophotometrically at 30 °C in the presence of 10 mM glycolate and 50–150 µg of soluble proteins using a coupled enzymatic assay comprising of o-dianisidine and horseradish peroxidase [25].

### 4.5. Multivariate and Statistical Analysis

Multivariate and statistical analyses were carried using R based [68] dedicated packages and personal scripts, except for the hierarchical clustering. Data normality was checked by visual inspection of quantile–quantile plots. Principal component analysis (PCA) was carried out with the R package FactoMineR [69] and the two first components showing the maximum of variability were used for graphical representations (variable plots with correlation circle and individual plots). Partial least squared discriminant analysis (PLS-DA) was performed with the mixOmics R package [70]. For hierarchical clustering, data were first visualized with a heat map after a normalization step and a mean-centered reduction step for each amino acid. Accordingly, the color scale in the heat map was expressed as the number of SDs (standard deviations). Hierarchical clustering based on Pearson correlation coefficients was achieved with the free Multiple Experiment Viewer software (MeV 4.9.0). The effect of genotype, [CO_2_] and their interaction were tested by a two-way ANOVA (*p*-value < 0.05). For amino acid analysis, the percentage of variance explained by the statistical model was calculated from the sum of squares (including residuals) and normality of the residues was checked with quantile–quantile plots. Tukey’s HSD tests were used for post-hoc multiple pairwise comparisons of mean groups and Student tests for the comparison of two mean groups (*p*-value < 0.05). All results are expressed as the mean ± standard deviation (SD) of three independent biological replicates.

## 5. Conclusions

Wild-type Arabidopsis and single *gox1 and gox2* mutants were compared to determine the impact of photorespiration on amino acid metabolism under low atmospheric CO_2_ concentrations. Increased photorespiration and reduced photosynthesis brought about by a reduction in CO_2_ levels rapidly led to a broad reconfiguration of leaf soluble amino acid pool sizes (Figure 1, Figure 2, Figure 3 and Figure 4; Tables S1 and S2). Low CO_2_ acclimation led to an accumulation of photorespiratory Gly and Ser (Figure 2B,C and Figure 4F,G; Tables S1–S4) and a significant reduction in Ala, Asp and Asn (Tables S1 and S2) whereas both Glu and Gln were less affected (Figure 4D; Tables S1 and S2). A perturbation of glyoxylate biosynthesis in *gox* mutants led to a further over-accumulation of Gly and Ser (Figure 4F,G) and this was negatively correlated to extractable GOX activity (Figure 3A). Metabolic flux studies and kinetic modelling of the interacting metabolic pathways are now required to better understand the processes involved in the low CO_2_-induced changes of leaf amino acid metabolism.

## Data Availability

The datasets for amino acid analysis are available in the “Supplementary Materials” section. The other datasets generated and/or analyzed during the current study are available from the corresponding authors on request due to restrictions.

## Supplementary Materials

The following are available online at https://www.mdpi.com/article/10.3390/metabo11080501/s1, Figure S1: Partial Least Square—Discriminant Analysis of rosette leaf amino acid contents of Arabidopsis Col-0 and *gox1* plants exposed to short-term low CO_2_ treatments (180 and 100 ppm for 4 h), Figure S2: Partial Least Square—Discriminant Analysis of rosette leaf amino acid contents of Arabidopsis Col-0, *gox1, gox2-1* and *gox2-2* plants exposed to long-term low CO_2_ treatment (200 ppm for 1, 4 or 8 days), Figure S3: Contribution of Gly and Ser to the variation of total amino acids after either short or long-term exposure to low CO_2_ treatment, Table S1: Complete dataset of free amino acid contents of Arabidopsis Col-0 and *gox1* rosette leaves exposed to short-term low CO_2_ treatments (180 and 100 ppm for 4 h), Table S2: Complete dataset of free amino acid contents of Arabidopsis Col-0, *go1x, gox2-1* and *gox2-2* plants exposed to long-term low CO_2_ treatments (200 ppm for 1, 4 or 8 days), Table S3: Free amino acid contents of rosette leaves from Arabidopsis Col-0 and *gox1* mutant exposed to short-term low CO_2_ treatments (180 and 100 ppm for 4 h) expressed as percentages of total amino acids, Table S4: Free amino acid contents of rosette leaves from Arabidopsis Col-0, *gox1* and *gox2* mutants exposed to long-term low CO_2_ treatment (200 ppm) for 1, 4 or 8 days expressed as percentages of total amino acids.
