# Supplementary material for: The Impact of Photorespiratory Glycolate Oxidase Activity on Arabidopsis thaliana Leaf Soluble Amino Acid Pool Sizes during Acclimation to Low Atmospheric CO2 Concentrations
Source: Metabolites. 2021 Jul 30;11(8):501. doi: 10.3390/metabo11080501 (PMC8399254; doi:10.3390/metabo11080501)
Supplement: Supplementary file 1 [file metabolites-11-00501-s001.zip › Figures S1-S3.pdf]

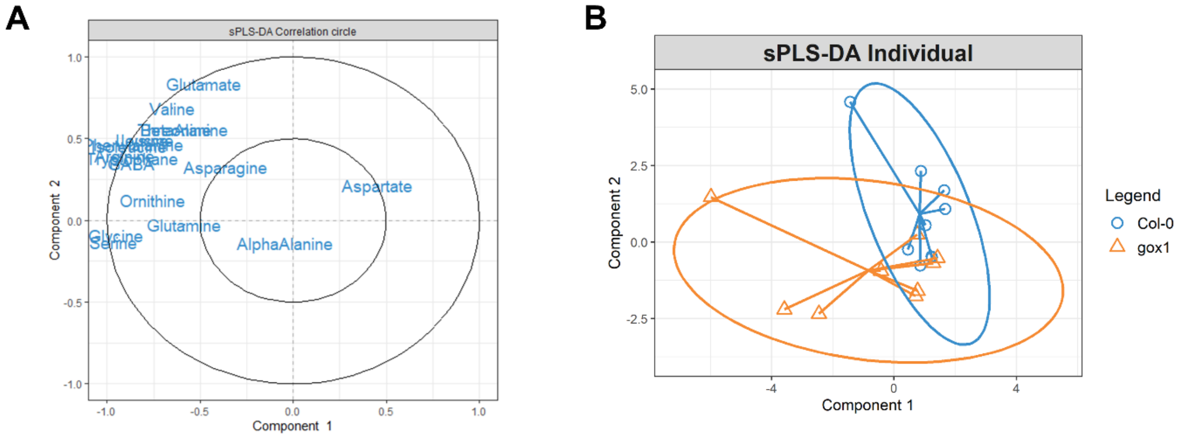

Figure S1. Partial Least Square – Discriminant Analysis for amino acid contents of rosette leaves from Arabidopsis Col-0 and *gox1* mutant exposed to short-term low CO_2 treatments (180 and 100 ppm for 4 h). (A) Variable (amino acids) plot with correlation circle and **(B)** Individual (Col-0 and *gox1*) plot**.**

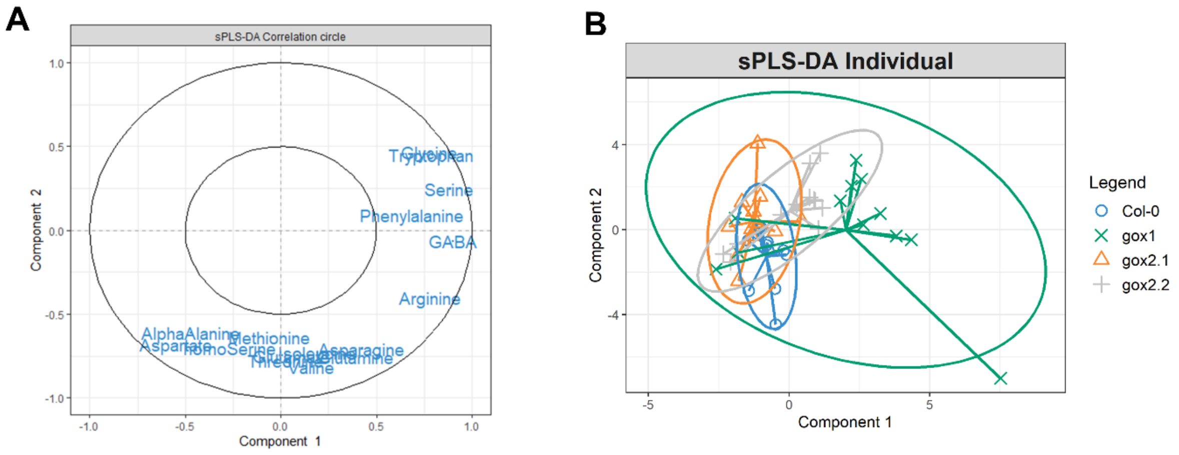

Figure S2. Partial Least Square – Discriminant Analysis for amino acid contents of rosette leaves from Arabidopsis Col-0, *gox1* and *gox2* mutants exposed to a long-term low CO_2 treatment (200 ppm for 1, 4 and 8 days). (A) Variable (amino acids) plot with correlation circle and **(B)** Individual (Col-0, *gox1*, *gox2.1*, *gox2.2*) plot**.**

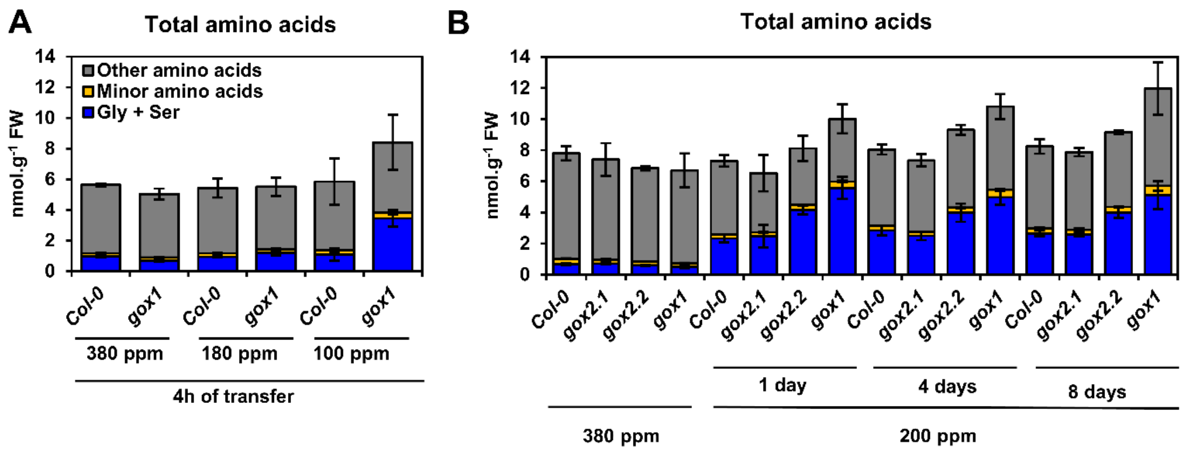

Figure S3. Contribution of Gly and Ser to the variation of total soluble amino acids after either short or long-term exposures to low CO_2. (A) Amino acid contents of rosette leaves of Arabidopsis Col-0 and *gox1* mutant exposed to short-term low CO_2 treatments (180 and 100 ppm for 4 h) and **(B)** Amino acid contents of rosette leaves of Arabidopsis Col-0, *gox1* and *gox2* mutants exposed to a long-term low CO_2 treatment (200 ppm for 1, 4 and 8 days). Minor amino acids were identified based on their very low contribution to the total soluble leaf amino acid pool (below <2%).
